# Accelerated Optical Projection Tomography Applied to *In Vivo* Imaging of Zebrafish

**DOI:** 10.1371/journal.pone.0136213

**Published:** 2015-08-26

**Authors:** Teresa Correia, Nicola Lockwood, Sunil Kumar, Jun Yin, Marie-Christine Ramel, Natalie Andrews, Matilda Katan, Laurence Bugeon, Margaret J. Dallman, James McGinty, Paul Frankel, Paul M. W. French, Simon Arridge

**Affiliations:** 1 Department of Computer Science, University College London, London, United Kingdom; 2 Division of Medicine, University College London, London, United Kingdom; 3 CoMPLEX, University College London, London, United Kingdom; 4 Department of Physics, Imperial College London, London, United Kingdom; 5 Department of Life Sciences, Division of Cell and Molecular Biology, Imperial College London, London, United Kingdom; 6 Department of Structural and Molecular Biology, University College London, London, United Kingdom; Pennsylvania State Hershey College of Medicine, UNITED STATES

## Abstract

Optical projection tomography (OPT) provides a non-invasive 3-D imaging modality that can be applied to longitudinal studies of live disease models, including in zebrafish. Current limitations include the requirement of a minimum number of angular projections for reconstruction of reasonable OPT images using filtered back projection (FBP), which is typically several hundred, leading to acquisition times of several minutes. It is highly desirable to decrease the number of required angular projections to decrease both the total acquisition time and the light dose to the sample. This is particularly important to enable longitudinal studies, which involve measurements of the same fish at different time points. In this work, we demonstrate that the use of an iterative algorithm to reconstruct sparsely sampled OPT data sets can provide useful 3-D images with 50 or fewer projections, thereby significantly decreasing the minimum acquisition time and light dose while maintaining image quality. A transgenic zebrafish embryo with fluorescent labelling of the vasculature was imaged to acquire densely sampled (800 projections) and under-sampled data sets of transmitted and fluorescence projection images. The under-sampled OPT data sets were reconstructed using an iterative total variation-based image reconstruction algorithm and compared against FBP reconstructions of the densely sampled data sets. To illustrate the potential for quantitative analysis following rapid OPT data acquisition, a Hessian-based method was applied to automatically segment the reconstructed images to select the vasculature network. Results showed that 3-D images of the zebrafish embryo and its vasculature of sufficient visual quality for quantitative analysis can be reconstructed using the iterative algorithm from only 32 projections—achieving up to 28 times improvement in imaging speed and leading to total acquisition times of a few seconds.

## Introduction

Optical projection tomography (OPT) is the optical analogue of X-ray computed tomography (CT) [1]. OPT can provide high (*μ*m) resolution three-dimensional (3-D) images of the optical attenuation (anatomy) and/or fluorescence intensity distribution within transparent samples. The technique entails wide-field imaging of the sample from different angles, typically using a scientific camera to sequentially capture the *projection image* at each angle. For transmission data, the sample should be illuminated along the optical axis of the projection, but for fluorescence OPT the direction of the illumination is immaterial and only reasonably uniform illumination throughout the sample is required. The sequential acquisition of each row of pixels of the camera provides a series of 1-D images as a function of the rotation angle to form a *sinogram*. Conventionally, each sinogram can be reconstructed using the filtered back projection (FBP) algorithm [2], which is based on the inverse Radon transform, to obtain a 2-D slice through the sample. Thus, reconstruction of all sinograms (slices) yields the whole 3-D sample volume.

OPT is widely applied to mm-cm scale transparent samples that have been optically cleared or are inherently transparent, including live organisms [3–10]. Zebrafish are currently attracting much interest as a model for development of diagnostic and therapeutic strategies relevant to variety of diseases, due to their ease of genetic manipulation, high fecundity, external and rapid embryo development, low maintenance costs, similarity to humans at molecular and cellular levels, and the ability to generate fluorophore labelled transgenic reporter lines [11]. For example, zebrafish can be used as a model for studying angiogenesis (the formation of new blood vessels from the existing vasculature), which plays a critical role in cancer progression and metastasis [12, 13]. Thus, zebrafish make an excellent *in vivo* vertebrate model and with transparent mutants, which lack genes for pigmentation, are well-suited to imaging using OPT. To date, most *in vivo* experiments have been conducted using medium to high resolution wide field and confocal microscopy in embryos up to 3 days post fertilisation (dpf), which are chemically modified to suppress pigmentation [14–16]. However, it is becoming apparent that developmental processes such as angiogenesis would benefit greatly from low magnification and high resolution whole animal *in vivo* imaging. In particular, OPT could provide a better understanding of certain disease processes and their causes, which may allow the improvement and development of diagnostic and therapeutic strategies. Here, we have used an optically transparent non-pigmented mutant line (TraNac) [15], expressing the vascular reporter gene (KDR:mCherry) to visualise the whole vascular network of a zebrafish embryo using OPT. Imaging juvenile/adult zebrafish *in vivo*, even the optically translucent non-pigmented lines (Casper/TraNac) [15, 16], is more challenging owing to the larger size and the increased impact of optical scattering.

For *in vivo* imaging, it is important to minimise the acquisition time and light dose, particularly if longitudinal studies are required. It is important to reduce the acquisition time since this reduces the time the zebrafish needs to be maintained under anaesthetic and it reduces the possibility that the zefrafish might move during the OPT acquisition. It is also desirable to reduce the light dose to reduce phototoxicity, which can present a challenge to live organisms, and to reduce photobleaching of the fluorescent proteins. The light dose and total acquisition time scale with the number of angular projections at which data is acquired. Theoretically, according to the classic Shannon-Nyquist sampling theorem, the number of projections or angular measurements necessary to accurately reconstruct the 3-D attenuation/fluorescence distribution with FBP should be proportional to the number of resolution elements in the projection image [2]. However, acquiring a complete data set can be very time consuming, requiring zebrafish embryos to be kept under anaesthesia for long periods of time and consequently less likely to recover, thus precluding longitudinal studies in the same zebrafish embryos. Furthermore, it is our intention to extend OPT imaging to juvenile/adult zebrafish and multiple fluorophores, therefore, it is desirable to reduce the acquisition time by decreasing the number of projections. However, using FBP leads to image degradation by streak artefacts when the number of projections is insufficient.

An alternative approach is to use compressed sensing (CS), which has gained much attention in the field of medical imaging, particularly magnetic resonance imaging (MRI) and CT, and several methods have been proposed to reduce scan times [17, 18]. The theory of CS suggests that if an image is sparse/compressible in some transform domain, it can be recovered from a reduced number of measurements [19], i.e., from fewer measurements than required by the Shannon-Nyquist sampling theorem. Many approaches use sparsity promoting regularisers, such as the commonly used total variation (TV), which promotes sparsity in the gradient domain and has edge-preserving properties. The TV regularisation method was originally proposed for image denoising [20], but subsequently TV minimisation algorithms have been developed for other applications, including image reconstruction from under-sampled data [21–27].

Here, for the first time to our knowledge, we implement a similar strategy for OPT in order to reduce the acquisition time/number of projections whilst retaining image quality. Using transmission and fluorescence projection images of a zebrafish embryo with fluorophore labelled vasculature, we demonstrate the ability of the method to reconstruct images from incomplete data sets.

## Materials and Methods

### Zebrafish sample preparation

Maintenance, breeding and use of animals conformed with UK ethical and licensing rules (UK Home Office PPL 70/7700). A completed ARRIVE (Animal Research: Reporting *In Vivo* Experiments) checklist can be found in S1 ARRIVE Checklist. The transparent TraNac zebrafish embryo used in this study is a double mutant for the *mpv17/transparent* and the *mitfa/nacre* genes (gift from Julian Lewis, Cancer Research UK, London Research Institute). A TraNac fish was crossed with a Tg(KDR:mCherry) fish (gift from Steve Wilson, University College London) to generate a TraNac Tg(KDR:mCherry) transparent transgenic line. The resultant fish is transparent with the vasculature labelled with mCherry. The embryo (4 dpf) was placed in a fluorinated ethylene propylene (FEP) tube with a 793 *μ*m inner diameter, which has a refractive index similar to water, filled with an anaesthetic solution (4.2% of a 4 mg/mL Tricaine solution). Agarose was added to increase the viscosity of the water and prevent movement of the anaesthetised zebrafish. Finally, the FEP tube was suspended in a cuvette filled with water, resulting in an approximately uniform refractive index environment.

### OPT imaging

The embryo was imaged using a simple OPT system similar to that described in [9], but implemented with the zebrafish mounted in a tube rotated about a vertical axis and suspended in a rectangular-sided cuvette filled with water. The imaging path for the OPT system comprised of a 4× Nikon objective and 200 mm focal length tube lens (N4X-PF and ITL200, Thorlabs Inc) mounted so that the imaging path was horizontal (see S1 Fig). An aperture was positioned directly behind the objective to reduce its effective numerical aperture to 0.04 and produce a depth of field of 0.6 mm, imaging the front half of the sample “in focus”. The tube-mounted sample was suspended beneath a stepper motor (T-NM17A200, Zaber Technologies Inc) and images were captured using a high resolution sCMOS cameras (Zyla 5.5, Andor Technology Ltd) with 2560 × 2160 pixels of 6.5 *μ*m × 6.5 *μ*m size. Transmission and fluorescence projection data were acquired at 800 evenly spaced angular positions over a full rotation, i.e. 360°(all data downloadable from http://dx.doi.org/10.6084/m9.figshare.1504041). The mCherry fluorophore was excited using a 561 nm laser (Jive^TM^, Cobolt AB) and imaged through a band pass filter centred at 641 nm (FF01-641/75-25, Semrock Inc). The total image acquisition time for each densely sampled data set (transmission or fluorescence) was about 1 min 50 s. The same process was repeated, using the same live zebrafish embryo, to acquire under-sampled data sets of 64, 50, 40, 32 and 20 projections, with total acquisition times of approximately 9 s, 7 s, 5 s, 4 s and 1 s, respectively. The number of projections was chosen to create evenly spaced steps in one revolution of the stepper motor. Note that acquisition time does not scale linearly with the number of projections, primarily because of the time required to save each projection image to disk. We also note that there is an additional overhead of 4 s required to initialise each image acquisition. The reconstructed 3-D volumes consisted of high resolution images of dimensions 981 × 981 × 2560.

### TV-regularised image reconstruction

The OPT forward model or projector is given by:

$$\begin{array}{c} Y=RX+n, \end{array}$$ (1)

where Y is the measured sinogram corrupted by noise *n*, R is the Radon transform and X is the (unknown) attenuation/fluorescence distribution. The inverse problem consists in recovering X from the noisy measurements Y, which for a complete data set can be achieved using FBP. Images can be reconstructed from incomplete measurements by solving the following optimisation problem:

$$\begin{array}{c} \underset{X}{\text{minimise}}\;\;\frac{1}{2}{∥Y-RX∥}_{2}^{2}+\;\tau \Phi_{\text{TV}}\left(X\right), \end{array}$$ (2)

where the first term is the *ℓ*_2_ norm of the residual, *τ* is the regularisation parameter and Φ_TV_(X) is the TV regularisation functional given by:

$$\begin{array}{c} \Phi_{\text{TV}}\left(\text{X}\right)\;=\;\sum_{i}\sqrt{{\left(D_{i}^{H}\right)}^{2}+{\left(D_{i}^{V}\right)}^{2}}, \end{array}$$ (3)

where *D* ^*H*^ and *D* ^*V*^ represent the first order finite difference (gradient) of X at pixel *i* in the horizontal and vertical direction, respectively.

The solution to the minimisation problem in Eq (2) can be obtained using the two-step iterative shrinkage/thresholding algorithm (TwIST) [28] (software can be found at http://www.lx.it.pt/∼bioucas/TwIST/TwIST.htm), which is defined as:

$$\begin{array}{c} {\text{X}}_{1}\;=\;\Gamma_{\tau }\left({\text{X}}_{0}\right) \end{array}$$ (4)

$$\begin{array}{c} {\text{X}}_{t+1}=\left(1-\alpha \right){\text{X}}_{t-1}+\left(\alpha -\beta \right){\text{X}}_{t}+\beta \Gamma_{\tau }\left({\text{X}}_{t}\right), \end{array}$$ (5)

for *t* ≥ 1, where *α* and *β* are constant parameters that depend on the eigenvalues of (R^T^R) and Γ_*τ*_ is given by:

$$\begin{array}{c} \Gamma_{\tau }(X)=\Psi_{\tau }(X+R^{T}\left(Y-RX\right)), \end{array}$$ (6)

where R^T^ is the back projection operator and Ψ_*τ*_ is the total variation denoising method, solved using the iterative scheme proposed by Chambolle [29].

Using FBP, we reconstructed the densely sampled transmission and fluorescence data sets, each comprising of 800 projection images, to provide ideal “reference”images. To explore the capabilities of the TwIST algorithm, we select subsets of 180, 90, 80, 70, 60, 50, 40, 30 and 20 evenly spaced projections from the densely sampled fluorescence data set and reconstructed images from these down-sampled data sets using the TwIST algorithm for different *τ* and TV minimisation iterations (TV_it_). Since the aim was to analyse the influence of these parameters on the quality of the reconstructions, the fluorescence data were used rather than the transmission images because these contain more detail (vasculature network). The parameters tested were *τ* = {0.002,0.006,0.01} and TV_it_ = {5, 20, 40}. These values were empirically selected, noting that lower *τ* values produce noisy images, whereas larger *τ* values result in over-smooth solutions, losing low contrast or detailed information, and larger TV_it_ do not significantly improve the reconstructions. It is desirable to keep the number of TV_it_ low, subject to obtaining a reconstructed image of satisfactory quality, since the computation time increases as the number of iterations increase. The remaining parameters were set at the default values [28]. Having established the optimal parameters for each down-sampled fluorescence data set, these parameters were used to reconstruct images from the corresponding down-sampled transmitted image data sets. Finally, 3-D images were reconstructed from under-sampled transmission and fluorescence data sets that were experimentally obtained with correspondingly shorter acquisition times.

### Vasculature segmentation

Segmentation of blood vessels is an important step for the detection of vascular abnormalities, which is an indicator for the progression and stage of angiogenesis-related diseases [12, 14]. In this work, an automated blood vessel segmentation using a multiscale Hessian-based vesselness measure [30] is applied to the 3-D images obtained from the fluorescence measurements. This method uses the three eigenvalues (∣*λ*_1_∣ ≤ ∣*λ*_2_∣ ≤ ∣*λ*_3_∣) of the Hessian matrix to determine the likelihood that tube-like structures, i.e. vessels, are present at multiple scales. The Hessian at voxel *v* = (*x*, *y*, *z*) and scale *σ* is defined as a convolution with derivatives of Gaussians:

$$\begin{array}{c} H\left(v,\sigma \right)=\sigma^{2}\text{X}\left(v\right)*\frac{\partial^{2}G_{\sigma }\left(v\right)}{\partial v^{2}} \end{array}$$ (7)

where X is the reconstructed image and 𝓖_*σ*_ is a Gaussian kernel with standard deviation *σ*. For an ideal tubular structure we have ∣*λ*_1_∣ ≈ 0, ∣*λ*_1_∣ ≪ ∣*λ*_2_∣ and ∣*λ*_2_∣ ≈ ∣*λ*_3_∣. The sign of *λ*_2_ and *λ*_3_ is an indicator of brightness/darkness, i.e. when vessels are bright tubular structures in a dark background they are both negative. The vesselness function discriminates tube-like structures from other structures [30]:

$$\begin{array}{c} V\left(\sigma \right)=\{\begin{array}{cc} 0 & \text{ if }\lambda_{2}>0\text{ or }\lambda_{3}>0\\ \left(1-\text{exp}\left(-\frac{R_{A^{2}}}{2a^{2}}\right)\right)\text{ exp}\left(-\frac{R_{B^{2}}}{2b^{2}}\right)\;\left(1-\text{exp}\left(-\frac{S^{2}}{2c^{2}}\right)\right) & \text{ otherwise} \end{array} \end{array}$$

where *a*, *b* and *c* are thresholds that control the sensitivity of the vesselness measures, which are essential to distinguish between tube-like and plate-like structures (𝓡_𝓐_), sphere-like (𝓡_𝓑_) and background (𝓢):

$$\begin{array}{c} R_{A}=\frac{|\lambda_{2}|}{|\lambda_{3}|}, \end{array}$$ (8)

$$\begin{array}{c} R_{B}=\frac{|\lambda_{1}|}{\sqrt{|\lambda_{2}\lambda_{3}|}}, \end{array}$$ (9)

$$\begin{array}{c} S=\sqrt{\lambda_{1}^{2}+\lambda_{2}^{2}+\lambda_{3}^{2}}. \end{array}$$ (10)

In this work, *a* = *b* = 0.5 and *c* = 500. The final estimate of vesselness at different scales is given by:

$$\begin{array}{c} V=\max_{\sigma_{min}\leq \sigma \leq \sigma_{max}}V(\sigma ), \end{array}$$ (11)

where *σ*_*min*_ and *σ*_*max*_ are the minimum and maximum scales, respectively.

The vesselness measure method was used to segment the blood vessels in the fluorescence images generated from the densely sampled and down-sampled data sets, as well as the under-sampled experimental data sets, using the optimal TwIST parameters.

### Assessment of image quality

The structural similarity index (SSIM) [31] was used to analyse the quality of the images reconstructed from reduced transmission and fluorescence data sets. The images obtained from the densely sampled data set using FBP are used as reference or “gold standard” G. The SSIM is defined as:

$$\begin{array}{c} \text{SSIM}\left(X,G\right)=\frac{\left(2\mu_{X}\mu_{G}+c_{1})(2\sigma_{\text{XG}}+c_{2}\right)}{\left(\mu_{X}^{2}+\mu_{G}^{2}+c_{1})(\sigma_{X}^{2}+\sigma_{G}^{2}+c_{2}\right)} \end{array}$$ (12)

where *μ*_X_ is the average of X, *μ*_G_ is the average of G, *σ*_X_ is the standard deviation of X, *σ*_G_ is the standard deviation of G and *σ*_XG_ is the covariance between X and G. The constants *c*_1_ and *c*_2_ are related to dynamic range of the images. If the SSIM is 1, it indicates that the two images are identical, and if the SSIM ≥ 0.9, they are considered to be highly similar.

The figure of merit used to analyse the quality of the segmented images was the Dice similarity index (DSI) [32], which is an overlap measure:

$$\begin{array}{c} \text{DSI}\left(X,G\right)=\frac{2|S_{X}\cap S_{G}|}{|S_{X}\left|+\right|S_{G}|} \end{array}$$ (13)

where S_X_ and S_G_ are segmented images obtained by applying the vesselness measure to the X and G images, respectively. A DSI value close to 1 indicates a perfect overlap between S_G_ and S_X_, whereas a value of 0 indicates no overlap. A DSI value ≥ 0.7 indicates an excellent agreement between the segmentation results and the segmented reference image and DSI values between 0.6 and 0.7 are considered to denote a substantial agreement between the two.

## Results and Discussion

### Algorithm parameters

**Fig 1:**
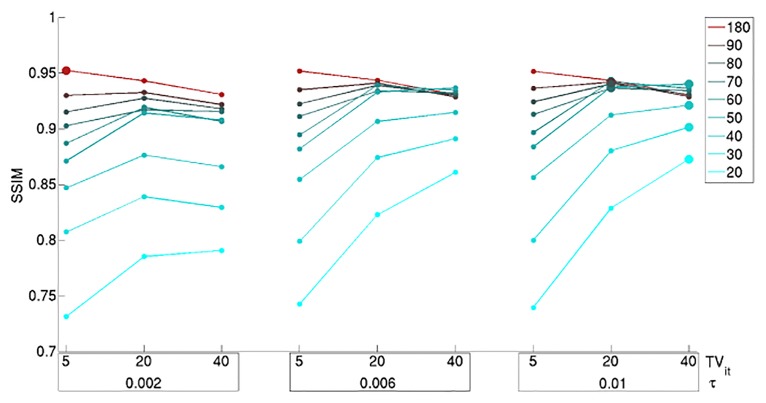
SSIM obtained for the images reconstructed from the down-sampled data sets using different algorithm parameters. The SSIM values obtained for different numbers of projections are represented by a different colour. The larger dots indicate the highest SSIM for each down-sampled data set and, hence, the optimal *τ* and TV_it_ parameters.

Fig 1 shows the SSIM obtained for the fluorescence images reconstructed from 180, 90, 80, 70, 60, 50, 40, 30 and 20 projections for different *τ* and TV_it_ values. The larger dots mark the highest SSIM for each data set and, hence, the parameters that generate the image with the highest quality. The highest SSIM for 180 projections was achieved using *τ* = 0.002 and TV_it_ = 5, for 90, 80, 70 and 60 projections using *τ* = 0.006 and TV_it_ = 40 and for 50, 40, 30 and 20 projections the optimal parameters were *τ* = 0.01 and TV_it_ = 40. Note that for 40 and more projections the SSIM does not fall below ∼ 0.85, indicating a very good similarity between the images reconstructed from the densely sampled and down-sampled data sets. The number of TV iterations seems to have a stronger influence on the quality of the reconstructions than the parameter *τ*. Nevertheless, this effect is only significant when using a small number of projections. The images obtained from the down-sampled data set consisting of 50 projections using different parameters are shown in S2 Fig. (see supplementary information) and are visually very similar. Therefore, if image reconstruction speed is preferred over image quality, we can use a small number of TV_it_ for a faster convergence of the algorithm, without noticeably reducing image quality.

### Image quality

**Fig 2:**
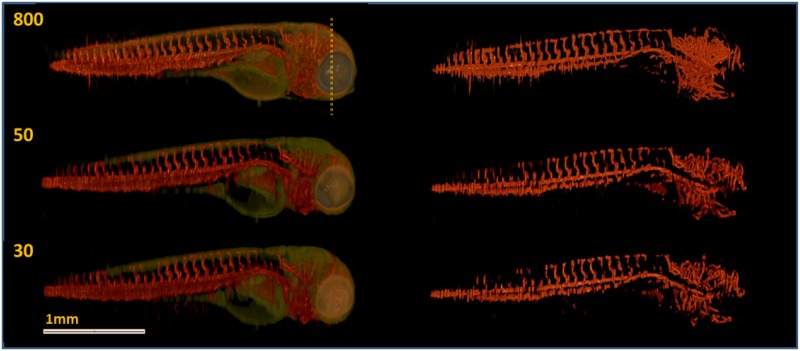
TraNac Tg(KDR:mCherry) zebrafish embryo images reconstructed from the densely sampled (800 projections) and down-sampled data sets of 50 and 30 projections. (Left) Images reconstructed from the fluorescence [red] and transmission projection images [green] and (right) corresponding segmentation images. The number of projections used to reconstruct each image is indicated in the corresponding subfigure (left). Note that the loss of resolution at the zebrafish tail is attributed to the live embryo moving its tail outside the depth of field of the imaging system during the experiment. Scale bar pertains to all images.

Fig 2 shows the image reconstructed from 800 fluorescence projection images superimposed on the reconstructed transmission projection image (referred to as anatomical image) and the corresponding segmentation image. The fluorescence, anatomical and segmentation images obtained from the down-sampled data sets consisting of 50 and 30 projections are also displayed in Fig 2. The images represent 3-D renderings from voxel values obtained using Drishti [33]. A high level of vasculature detail is retained in the images reconstructed from at least 50 fluorescence projection images and corresponding segmented images, particularly in the head region (Fig 2). All the reconstructed images from transmission and fluorescence projections are shown in S3 Fig. Note that there is a significant amount of noise in the images obtained from the densely sampled data set, which results in a denser vasculature appearance (Fig 2). The vasculature detail in the tail and head is preserved in images reconstructed from down-sampled data sets of 50 or more projections. Moreover, the head vasculature detail is recovered using as little as 30 projections (Fig 2). All the segmented images are shown in S4 Fig. The segmentation method extracts blood vessels of diameter greater than ∼20 *μ*m (which is the majority) and, hence, we believe this approach is useful to visualize and quantify the main features of the vasculature structure of zebrafish.

**Fig 3:**
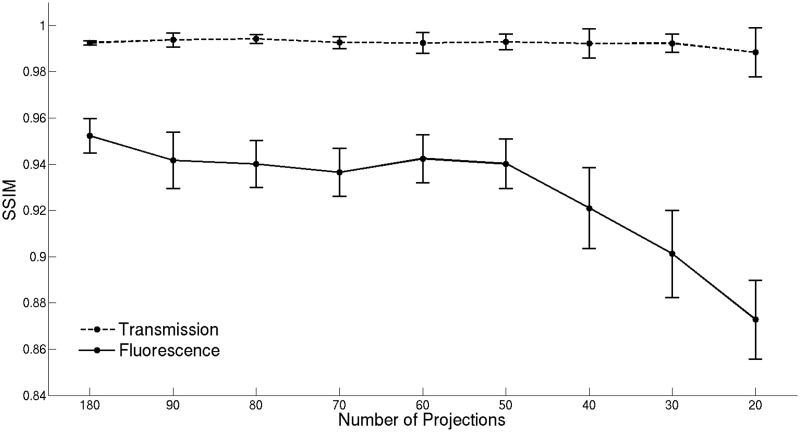
SSIM obtained for the images reconstructed from the down-sampled data sets. The plots show the SSIM for the images reconstructed from transmission and fluorescence measurements using different numbers of projection images. Error bars represent the maximum absolute error estimated for the images obtained from each down-sampled data set.

**Fig 4:**
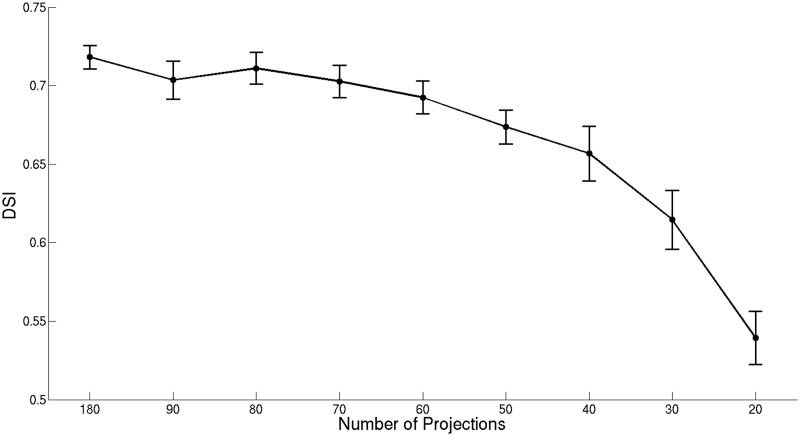
DSI obtained for the segmented vasculature images. The plot shows the DSI for the segmentation obtained from the images reconstructed from the down-sampled data sets using different numbers of projection images. Error bars represent the maximum absolute error estimated for the images obtained from each down-sampled data set.

Fig 3 shows the SSIM calculated for the images reconstructed from the down-sampled transmission data sets and the best results obtained for the fluorescence images, corresponding to the larger dots in Fig 1. The results indicate that the quality of anatomical images obtained from transmission measurements is preserved, with the SSIM being greater than 0.98 even when the number of projections is as low as 20. This is because the transmission images are dominated by large anatomical features of high contrast (e.g. the eyes) that are preserved without significant degradation in the reconstructed images as the number of projections is reduced. The SSIM values indicate a high similarity between the images reconstructed from 30 or more fluorescence projections and the reference image obtained from 800 projections. Note that the SSIM values do not change significantly for 50, 60, 70, 80, 90 and 180 projections, meaning that the quality of the reconstructed vasculature images is similar. It was not possible to obtain useful reconstructions for down-sampled data sets with 10 or fewer projections. The DSI values obtained after segmentation are displayed in Fig 4. The DSI values show that there is a substantial agreement between the segmented reference image and the segmented vasculature images obtained from the down-sampled data sets consisting of 30 or more projections. Moreover, the DSI values indicate that the segmentation is particularly effective when 70 or more projections are used to obtain the segmented vasculature images. Note that the SSIM and DSI values obtained are associated with an estimated statistical uncertainty (indicated by the error bars in Figs 3 and 4), which was determined by performing multiple reconstructions for each number of projections but randomly selecting different angular projections from the full data set.

### Comparison with the FBP method

**Fig 5:**
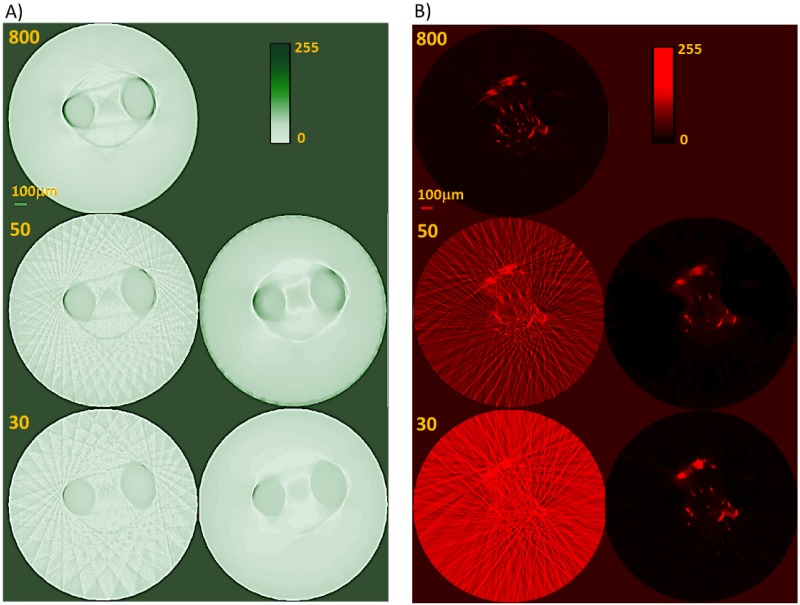
TraNac Tg(KDR:mCherry) zebrafish embryo 2D transaxial slices reconstructed from the densely sampled (800 projections) and down-sampled data sets of 50 and 30 projections using the conventional FBP method and iterative image reconstruction strategy. Zebrafish head slices reconstructed (A) from the transmission projection images using (left) the FBP algorithm and (right) the iterative image reconstruction strategy. (B) Slices reconstructed from the fluorescence projection images using (left) the FBP algorithm and (right) the iterative image reconstruction strategy. The number of projections used to reconstruct the images is indicated in each subfigure (left). Reconstructed slices are presented in a 0–255 contrast scale as indicated by the color bars. Scale bar pertains to all images.

For comparison, images were also reconstructed from the down-sampled data sets using the conventional FBP method. Streak artefacts appear in the images due to the insufficient number of projections, which compromise image quality and obscure vascular and anatomical structural details. Indicative results are displayed in Fig 5 as 2D transaxial image slices of the zebrafish head region (location indicated with a dashed line in Fig 2, top left). Fig 5A and 5B show the slices reconstructed with FBP using 800, 50 and 30 transmission and fluorescence projection images, respectively. The slices obtained from the down-sampled data sets of 50 and 30 projections using the iterative reconstruction method are also displayed in Fig 5. The streak artefacts are clearly visible in the images reconstructed using the FBP algorithm due to insufficient angular sampling. As expected, these become more pronounced as the number of projections decreases. The iterative image reconstruction strategy performs much better than the conventional FBP method effectively reducing the image artefacts. This is particularly striking in the fluorescence reconstructions using 30 projections (Fig 5B); the FBP method produces images that are visually very poor, whereas the iterative method recovers most of the vasculature detail.

**Fig 6:**
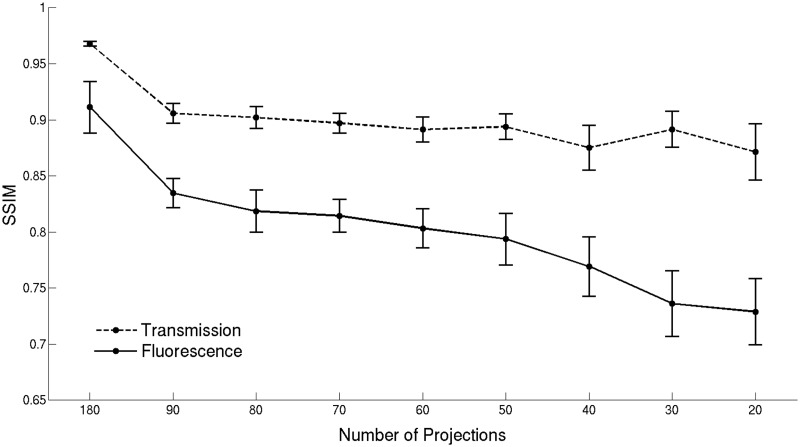
SSIM obtained for the images reconstructed from the down-sampled data sets using the FBP method. The plots show the SSIM for the images reconstructed from transmission and fluorescence measurements using different numbers of projection images. Error bars represent the maximum absolute error estimated for the images obtained from each down-sampled data set.

Fig 6 shows the SSIM obtained for the volumetric images reconstructed with FBP using 180, 90, 80, 70, 60, 50, 40, 30 and 20 transmission and fluorescence projections. We note that the quality of the anatomical images obtained from transmission measurements does not degrade as significantly as for the fluorescence images because the former are dominated by large high contrast features that are easier to recover than small structures. Nevertheless, the image quality does decrease with decreasing number of projections due to the increasingly prominent streak artefacts. The SSIM of the images reconstructed from 180 transmission and fluorescence projections with FBP is higher than 0.9, indicating high similarity to the images obtained from the densely sampled data set with 800 projections. Nonetheless, the iterative method still provides images of superior quality and SSIM. The transaxial slices reconstructed from all the down-sampled transmission and fluorescence data sets with FBP are shown in S5 and S6 Figs, respectively. The corresponding reconstructions using the iterative strategy are shown in S7 and S8 Figs. Thus, these results indicate that the iterative image reconstruction method has a better performance than FBP, particularly in suppressing streak artefacts in the reconstructed images, generating images from down-sampled data sets that are visually and quantitatively more accurate than those obtained with FBP. These streak artefacts lead to errors in the segmentation process that make under-sampled FBP unsuitable for this purpose.

### 3-D reconstruction of a zebrafish embryo

**Fig 7:**
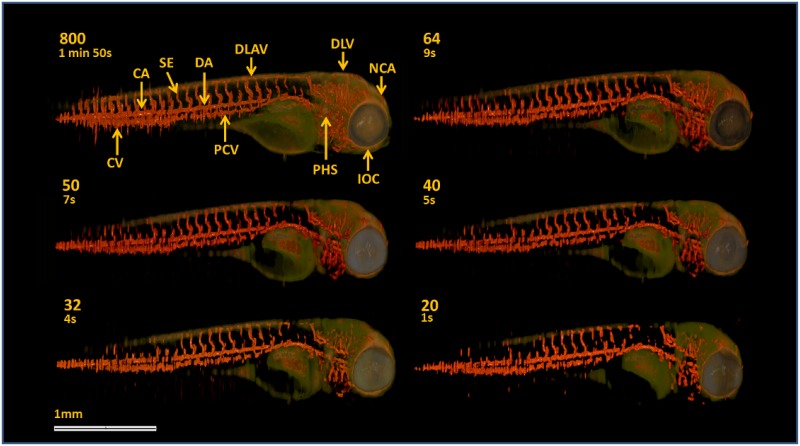
TraNac Tg(KDR:mCherry) zebrafish embryo anatomical [green] and segmented vasculature images [red] obtained from the under-sampled data sets. Anatomical and segmented vasculature images obtained from the transmission and fluorescence under-sampled data sets. The number of projections used to reconstruct the images and acquisition time is indicated in each subfigure. The vasculature labels (top left) indicate some of the key features of the zebrafish embryo vasculature: caudal vein (CV), caudal artery (CA), intersegmental vessel (SE), dorsal aorta (DA), posterior cardinal vein (PCV), dorsal longitudinal anastomotic vessel (DLAV), primary head sinus (PHS), dorsal longitudinal vein (DLV), inner optic circle (IOC) and nasal ciliary artery (NCA). Scale bar applies to all panels.

Fig 7 shows the images reconstructed from the experimentally generated under-sampled data sets, consisting of 64, 50, 40, 32 and 20 segmented vasculature images superimposed on the corresponding anatomical images. For comparison, the images obtained from 800 projections using FBP are also displayed in Fig 7. As indicated in Fig 7, using OPT we can identify the key vascular structures corresponding to a 4 dpf embryo [34]. Note that even though 800 projections does satisfy the angular sampling theorem for this OPT system, the measurement noise in individual projection images and the fish moving its tail outside the depth of field can still produce artefacts in the FBP reconstruction. The images obtained from 64 and 50 projections exhibit high vasculature detail, particularly in the head region, comparable to that obtained using 800 projections, with the advantage of being about 12 and 16 times faster to acquire data, respectively. The images deteriorate for fewer projections. Nevertheless, a large number of blood vessels are still visible in the images, including those obtained from 40 and 32 projections for which the acquisition times are about 22 and 28 times faster, respectively.

**Fig 8:**
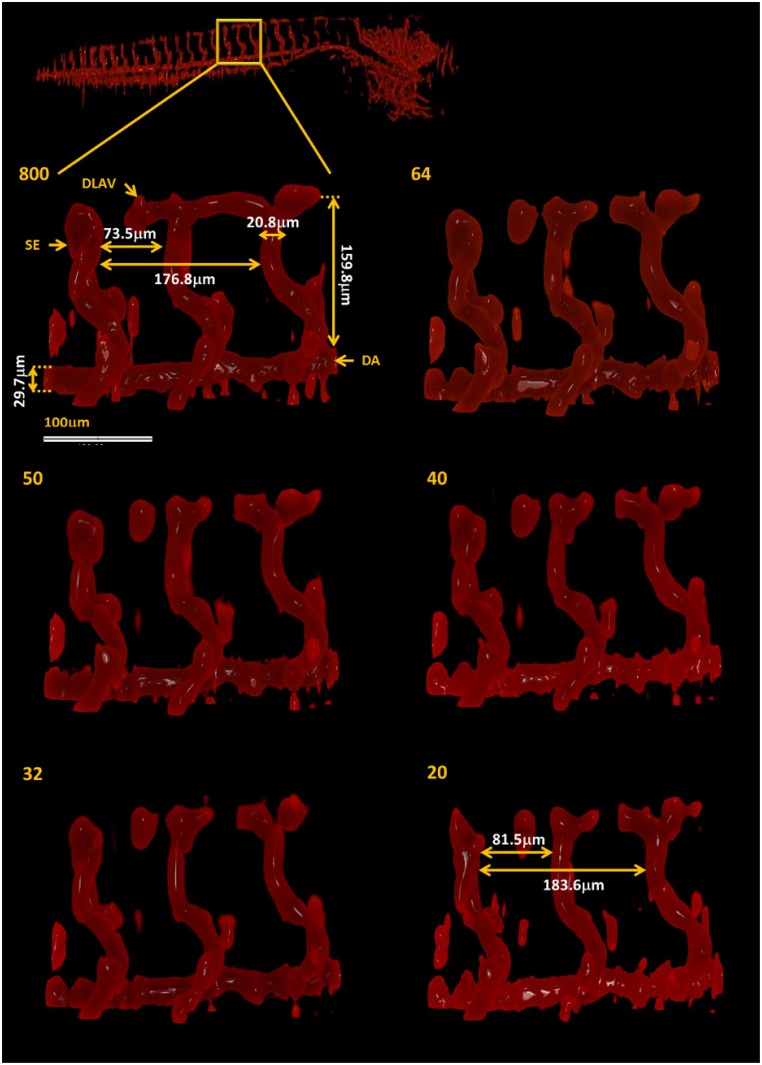
TraNac Tg(KDR:mCherry) zebrafish embryo magnified views of the segmented vasculature images obtained from the under-sampled data sets. The box indicates the region magnified (top left) The number of projections used to reconstruct the vasculature images is indicated in each subfigure (left). The vasculature labels indicate the intersegmental vessel (SE), dorsal aorta (DA) and dorsal longitudinal anastomotic vessel (DLAV). Scale bar applies to all magnified views.

Fig 8 shows magnified views of the segmented vasculature images in Fig 7, which were obtained from the fully sampled and under-sampled data sets ranging from 64 to 20 projections, corresponding to the areas indicated by the box. The magnified view of the image obtained using 800 projection presents some of the key features of the zebrafish vasculature and can provide quantitative information, such as vessel thickness and distance between vessels. Apart from some small sections of the dorsal longitudinal anastomotic vessel (DLAV), these features are preserved in the segmented vasculature images obtained from the under-sampled data sets with remarkable fidelity. For example, the thickness of the intersegmental vessel (SE) and dorsal aorta (DA), the apparent distance between SEs and between the DA and DLAV do not change significantly for the images obtained from 64, 50, 40 and 32 projections. These features are still recovered using as few as 20 projections, but the distance between SEs slightly increases (less than 10 *μ*m). The transgenic zebrafish model imaged here is routinely used to determine the effects of gene manipulation and/or drug treatment on angiogenesis. It is important to note that the vascular images and corresponding dimensions shown in Figs 7 and 8 are in agreement with those presented in the literature and are of sufficient quality to determine changes in angiogenesis [35, 36].

The maximum intensity projection method (MIP) was used to generate 2-D images from the 3-D fluorescence reconstruction and segmentation images, obtained from 800, 50 and 32 (under-sampled) projections, by projecting the voxel with maximum intensity, along each projection ray, onto a 2-D image at several viewing angles. The 2-D MIP images were used to produce the 3-D animations in the Supporting Information Section (see S1–S6 Videos).

## Conclusions

Long OPT data acquisition times limit the potential to perform *in vivo* longitudinal studies in zebrafish and images are more prone to be affected by undesirable motion artefacts, whereas shorter acquisitions result in reduced image quality. We have demonstrated the use of an iterative image reconstruction method to obtain high quality images from under-sampled OPT data sets of a zebrafish embryo, including transmitted and fluorescence projection measurements. We have obtained high quality images using as few as 50 projections, i.e., 6% of the densely sampled data set, which can reduce the total acquisition time by up to 16 times. Moreover, images of sufficient quality to discern blood vessels of thickness and separation on a scale of tens of *μ*m can be obtained from only 32 projections (4% of the densely sampled data set), enabling up to 28 times faster acquisition time. This approach will also be valuable when imaging larger zebrafish.

## Supporting Information

S1 Fig Photograph showing the OPT system. (EPS)

S2 Fig TraNac Tg(KDR:mCherry) zebrafish embryo images reconstructed from the down-sampled data set consisting of 50 fluorescence projections [red] using different algorithm parameters. Images reconstructed using (top) *τ* = 0.002, (middle) *τ* = 0.006 and (bottom) *τ* = 0.01 and (left) 5, (centre) 20 and (right) 40 TV iterations. The image that resulted in the highest SSIM is marked by the red square. However, no major differences are observed between the images. The transmission volumetric image [green] is displayed to provide an anatomical reference. Scale bar pertains to all images.
(EPS)

S3 Fig TraNac Tg(KDR:mCherry) zebrafish embryo anatomical [green] and vasculature [red] images reconstructed from the down-sampled data sets. The number of projections used to reconstruct the images is indicated in each subfigure. The quality of the images obtained from 800 projection with FBP is visually similar to those obtained with TwIST from at least 50 projections. Nevertheless, a large part of the vasculature network is still visible in the images obtained from less than 50 projection images. Scale bar pertains to all images.
(EPS)

S4 Fig TraNac Tg(KDR:mCherry) zebrafish embryo segmented vasculature [red] obtained from the images reconstructed using the down-sampled data sets. The number of projections used to obtain the vasculature images is indicated in each image. The large majority of the blood vessels were detected by the segmentation algorithm. The transmission volumetric image [green] is displayed to provide an anatomical reference.
(EPS)

S5 Fig TraNac Tg(KDR:mCherry) zebrafish embryo 2D transaxial slices reconstructed from the densely sampled (800 projections) and down-sampled transmission data sets using the conventional FBP method. The number of projections used to reconstruct the images is indicated in each subfigure (left). Reconstructed slices are presented in a 0–255 contrast scale as indicated by the color bars. Scale bar pertains to all images.
(EPS)

S6 Fig TraNac Tg(KDR:mCherry) zebrafish embryo 2D transaxial slices reconstructed from the densely sampled (800 projections) and down-sampled fluorescence data sets using the conventional FBP method. The number of projections used to reconstruct the images is indicated in each subfigure (left). Reconstructed slices are presented in a 0–255 contrast scale as indicated by the color bars. Scale bar pertains to all images.
(EPS)

S7 Fig TraNac Tg(KDR:mCherry) zebrafish embryo 2D transaxial slices reconstructed from the densely sampled (800 projections) and down-sampled transmission data sets using the iterative reconstruction method. The number of projections used to reconstruct the images is indicated in each subfigure (left). Reconstructed slices are presented in a 0–255 contrast scale as indicated by the color bars. Scale bar pertains to all images.
(EPS)

S8 Fig TraNac Tg(KDR:mCherry) zebrafish embryo 2D transaxial slices reconstructed from the densely sampled (800 projections) and down-sampled fluorescence data sets using the iterative reconstruction method. The number of projections used to reconstruct the images is indicated in each subfigure (left). Reconstructed slices are presented in a 0–255 contrast scale as indicated by the color bars. Scale bar pertains to all images.
(EPS)

S1 Video TraNac Tg(KDR:mCherry) zebrafish embryo 3-D MIP rotating animation representing the fluorescence reconstructions obtained from 800 projections. (AVI)

S2 Video TraNac Tg(KDR:mCherry) zebrafish embryo 3-D MIP rotating animation representing the segmented vasculature obtained from 800 projections. (AVI)

S3 Video TraNac Tg(KDR:mCherry) zebrafish embryo 3-D MIP rotating animation representing the fluorescence reconstructions obtained from the under-sampled data set of 50 projections. (AVI)

S4 Video TraNac Tg(KDR:mCherry) zebrafish embryo 3-D MIP rotating animation representing the segmented vasculature obtained from the under-sampled data set of 50 projections. (AVI)

S5 Video TraNac Tg(KDR:mCherry) zebrafish embryo 3-D MIP rotating animation representing the fluorescence reconstructions obtained from the under-sampled data set of 32 projections. (AVI)

S6 Video TraNac Tg(KDR:mCherry) zebrafish embryo 3-D MIP rotating animation representing the segmented vasculature obtained from the under-sampled data set of 32 projections. (AVI)

S1 ARRIVE Checklist Completed ARRIVE Checklist. (PDF)
